# Intermediate monocytes correlate with CXCR3^+^ Th17 cells but not with bone characteristics in untreated early rheumatoid arthritis

**DOI:** 10.1371/journal.pone.0249205

**Published:** 2021-03-26

**Authors:** Christina Drevinge, Julia M Scheffler, Catalin Koro-Arvidsson, Daniel Sundh, Hans Carlsten, Inger Gjertsson, Catharina Lindholm, Mattias Lorentzon, Anna Rudin, Anna-Karin Hultgård Ekwall, Ulrika Islander

**Affiliations:** 1 Centre for Bone and Arthritis Research, Institute of Medicine, Department of Internal Medicine and Clinical Nutrition, Sahlgrenska Academy, University of Gothenburg, Gothenburg, Sweden; 2 Krefting Research Center, Department of Internal Medicine and Clinical Nutrition, Institute of Medicine, Sahlgrenska Academy, University of Gothenburg, Gothenburg, Sweden; 3 Centre for Bone and Arthritis Research, Department of Rheumatology and Inflammation Research, Institute of Medicine, Sahlgrenska Academy, University of Gothenburg, Gothenburg, Sweden; 4 Geriatric Medicine, Department of Internal Medicine and Clinical Nutrition, Institute of Medicine, Sahlgrenska Academy, University of Gothenburg, Gothenburg, Sweden; 5 Department of Rheumatology and Inflammation Research, Institute of Medicine, Sahlgrenska Academy, University of Gothenburg, Gothenburg, Sweden; 6 Mary McKillop Institute for Health Research, Australian Catholic University, Melbourne, Australia; Mayo Clinic Rochester, UNITED STATES

## Abstract

**Background:**

Rheumatoid arthritis (RA) is associated with development of generalized osteoporosis. Bone-degrading osteoclasts are derived from circulating precursor cells of monocytic lineage, and the intermediate monocyte population is important as osteoclast precursors in inflammatory conditions. T cells of various subsets are critical in the pathogenesis of both RA and associated osteoporosis, but so far, no studies have examined associations between circulating intermediate monocytes, T cell subsets and bone characteristics in patients with RA. The aim of this study was to investigate the frequency of intermediate monocytes in patients with untreated early rheumatoid arthritis (ueRA) compared to healthy controls (HC), and to explore the correlation between intermediate monocytes and a comprehensive panel of T helper cell subsets, bone density and bone microarchitecture in ueRA patients.

**Methods:**

78 patients with ueRA fulfilling the ACR/EULAR 2010 criteria were included and compared to 29 age- and sex-matched HC. Peripheral blood samples were obtained before start of treatment and proportions of monocyte subsets and CD4^+^ helper and regulatory T cell subsets were analyzed by flow cytometry. Bone densitometry was performed on 46 of the ueRA patients at inclusion using DXA and HR-pQCT.

**Results:**

Flow cytometric analyses showed that the majority of ueRA patients had frequencies of intermediate monocytes comparable to HC. The intermediate monocyte population correlated positively with CXCR3^+^ Th17 cells in ueRA patients but not in HC. However, neither the proportions of intermediate monocytes nor CXCR3^+^ Th17 cells were associated with bone density or bone microarchitecture measurements.

**Conclusions:**

Our findings suggest that in early RA, the intermediate monocytes do not correlate with bone characteristics, despite positive correlation with circulating CXCR3^+^ Th17 cells. Future longitudinal studies in patients with longer disease duration are required to fully explore the potential of intermediate monocytes to drive bone loss in RA.

## Introduction

Rheumatoid arthritis (RA) is a systemic autoimmune disease characterized by joint pain and inflammation, which results in progressive destruction of cartilage and underlying bone [1,2]. In addition to periarticular bone loss of affected joints, RA is also associated with development of generalized osteoporosis resulting from an imbalance between the bone-resorbing osteoclasts and bone-forming osteoblasts [3,4].

The life span of osteoclasts is estimated to a few weeks and the osteoclast population is replenished by precursor cells of monocytic lineage circulating in peripheral blood [5]. Three monocyte subpopulations have been defined in humans based on differential expression of the lipopolysaccharide (LPS) receptor CD14 and the FcγIII receptor CD16 [6]. Classical monocytes [CD14^++^CD16^−^] is the most abundant subset during healthy conditions and has been shown to replenish the population of tissue resident peripheral monocyte-derived cells [7–9]. The classical monocytes are proposed to egress from the bone marrow to the circulation and evolve into non-classical monocytes [CD14^+^CD16^++^] via an intermediate monocyte subset [CD14^++^CD16^+^] [10]. The non-classical monocyte population is suggested to function as vascular housekeepers by patrolling the blood vessels [11], while the intermediate monocyte population was most recently described and has attracted interest because of its potential pathological role in infectious and inflammatory conditions [12,13]. Although osteoclasts can be derived from all three monocyte subsets under healthy conditions [14], findings implicate an important role for intermediate monocytes as osteoclast precursors in inflammatory diseases [15]. Monocytes from patients with inflammatory diseases associated with bone loss have a higher ability to form osteoclasts compared to monocytes from HC [16–18], and increased frequencies of the intermediate monocyte subset have been reported in peripheral blood from patients with RA [19–22]. However, in those studies the patient groups were not homogenous with regard to RA disease duration or treatment, indicating the need to study the monocyte subsets in a well-defined more homogenous group of RA patients. Furthermore, it still remains to be determined whether the intermediate monocyte subset is associated with decreased bone density or bone microarchitecture in RA patients.

The specific mechanisms involved in the pathological processes of RA remains unclear, but T cells of various subsets have been shown to play an important role. IL-17 producing CD4^+^ T helper cells (Th17) increases joint inflammation in RA *e*.*g*. through recruitment of neutrophils [23]. Furthermore, Th17 cells are one of the major actors in osteoclast development [24]. The cytokines M-CSF and RANKL are the most important factors driving the differentiation of monocytes to osteoclasts [25] and Th17 cells induces osteoclast differentiation by production of RANKL, as well as induction of M-CSF and RANKL expression by osteoblasts and stromal cells, and RANK expression on osteoclast precursors [26,27]. Previously, Th17 cells have been reported to correlate with the intermediate monocyte population in RA patients [19]. However, the relation between circulating intermediate monocytes and other CD4^+^T cell subtypes in blood from patients with RA is not known.

The differentiation to osteoclasts is also influenced by a complex milieu of other cells, cytokines, hormones and growth factors adjacent to the bone [28]. Chemokines and their corresponding surface bound receptors are involved in RA pathogenesis via regulation of immune cell trafficking and cell homing within tissues. The chemokine CXCL10 and its receptor CXCR3 are abundant in peripheral blood and local inflamed joints of patients with RA [29–32]. CXCL10 have also been reported to associate with disease activity in RA patients [33] and blocking of CXCR3 inhibits inflammatory cell infiltration and bone destruction in the joints of mice with collagen-induced arthritis by shifting the Th17/Treg cell balance [34]. In line with this, a previous study showed that expression of CXCR3 on T cells has an essential role in T cell recruitment to inflamed joints [35].

In this study, we investigated the role of monocytes as osteoclast progenitors and their correlation with bone density and bone microarchitecture in treatment-naïve patients with newly diagnosed RA. Also, we used multivariate analysis to examine the association between monocytes and a comprehensive T cell subset panel in peripheral blood of the untreated early RA (ueRA) patients. We demonstrate that the frequency of circulating intermediate monocytes is not significantly increased in patients with ueRA compared to HC. The intermediate monocyte population correlate positively with CXCR3^+^ Th17 cells in ueRA patients but not in HC. However, neither the proportions of intermediate monocytes nor CXCR3^+^ Th17 cells are associated with bone density or bone microarchitecture measurements. Thus, our results suggest that in early RA, the intermediate monocytes do not correlate with bone characteristics, despite positive correlation with circulating CXCR3^+^ Th17 cells.

## Materials and methods

### Study population

Seventy-eight patients with untreated early diagnosed RA (ueRA) fulfilling the American College of Rheumatology/European League Against Rheumatism 2010 criteria were included in the study and compared to a group of 29 age‐ and sex‐matched healthy controls (HC). Exclusion criteria for the HC were: ongoing acute disease, chronic disease, ongoing medication that suppresses inflammation or affects the immune system.

Clinical characteristics of ueRA patients and HC are shown in Table 1. The inclusion criteria were: ≥18 years old, ≥2 swollen joints and ≥2 tender joints, rheumatoid factor (RF)-positive or anti-citrullinated protein antibody (ACPA) positive or C reactive protein (CRP) ≥ 10 mg/ml, at least moderate disease activity (>3.2) measured by composite index disease activity score (DAS28)‐CRP, symptom duration <24 month (retrospective patient‐reported pain in the joints), and no treatment with corticosteroids or disease modifying anti-rheumatic drugs (DMARDs). The patients were included in the study from 2013 to 2018 and blood samples were taken within 1–2 weeks after RA diagnosis. The patients were recruited at the rheumatology clinic at Sahlgrenska University Hospital in Gothenburg, or at the rheumatology clinic at Skåne University hospital in Malmö and Lund. The study was approved by the regional ethics committees of Gothenburg and Lund, Sweden. All patients signed an informed consent form.

**Table 1:** Clinical characteristics of ueRA patients and HC.

	ueRA patients (*n* = 78)	HC (*n* = 29)	
Age, years	56 (21–80)		58 (20–75)
Female, n (%)	55 (71.0)		17 (58.6)
Self-reported symptom duration, months	5 (1–23)		NA
CRP, mg/L	9.3 (0.3–180)		NA
ESR, mm/hour	26 (5–120)		NA
SJC66	11 (3–30)		NA
TJC68	13.5 (2–47)		NA
SJC28	8 (2–24)		NA
TJC28	9 (0–27)		NA
DAS28-CRP	5 (2.7–8.3)		NA
DAS28-ESR	5.3 (2.6–8.7)		NA
CDAI	28.1 (10.1–68.7)		NA
ACPA+, n (%)	65 (83.3)		NA
RF+, n (%)	57 (73.1)		NA
ACPA+ and RF+, *n* (%)	52 (66.7)		NA
ACPA- and RF-, *n* (%)	8 (10.3)		NA
Smoker, n (%)^a^	11 (14.1)		NA

Continuous data is presented as median (range). *ACPA* anti-citrullinated protein/peptide antibodies, *CDAI* clinical disease activity index, *CRP* C-reactive protein, *DAS28* disease activity score in 28 joints, *ESR* erythrocyte sedimentation rate, *RF* rheumatoid factor, *SJC 28/66* swollen joint counts of 28/66, *TJC 28/68* tender joint counts of 28/68, arthritis, *NA* not analyzed. ^a^Current daily smoker.

### Clinical evaluation

Disease activity in patients was assessed by: swollen joint count of 66 joints (SJC 66), tender joint counts of 68 joints (TJC 68), swollen joint count in 28 joints (SJC 28), tender joint count in 28 joints (TJC 28), CRP, erythrocyte sedimentation rate (ESR), DAS28-CRP, DAS28-ESR and Clinical Disease Activity Index (CDAI). ACPA positivity was determined by multiplexed anti-CCP test (BioPlex from BioRad, Hercules, CA, USA) and RF positivity was determined by nephelometry (Beckman Coulter, Brea, CA, USA). Patients with >3 IU/ml anti-CCP antibodies or >20 IU/ml RF in serum were considered ACPA or RF-positive, respectively, according to the current cut-off levels in the clinical immunology laboratories.

### Flow cytometry

Peripheral blood samples were analyzed by flow cytometry. PBMCs were separated from whole blood with Lymphoprep (Axis‐Shield, Oslo, Norway). The cells were blocked with mouse serum and human AB serum. To define monocyte subsets the cell surface of PBMCs was stained with fluorochrome‐conjugated mAbs antibodies: APC-conjugated anti-CD14 (clone M5E2; BD Biosciences) and FITC-conjugated anti-CD16 (clone NKP15; BD Biosciences). Monocyte subsets were defined according to the gating strategy shown in **S1 Fig**. Initially monocytes were gated according to their forward scatter (FSC-Area) and side scatter (SSC-Area) characteristics. Doublet discrimination was done using FCS-Area and FCS-Height. The monocyte population was then subdivided based upon their expression of CD14 and CD16. Three monocyte subsets were distinguished: classical monocytes (CD14^++^CD16^-^), intermediate monocytes (CD14^++^CD16^+^) and non-classical monocytes (CD14^+^CD16^++^) as previously described [6]. T cells were stained and defined as previously described [36]. Briefly, for surface staining the following antibodies were used: FITC‐conjugated anti-CD45RA (clone L48; BD Biosciences) and anti-CD127 (clone HIL‐7R‐M21; BD Biosciences); PE‐conjugated anti-CCR6 (clone G034E3; Biolegend, San Diego, CA, USA); APC‐H7‐conjugated anti-CD4 (clone SK3; BD Biosciences); APC/AF647‐conjugated anti-CD127 (clone HIL‐7R‐M21; BD Biosciences), anti-CXCR5 (clone RF8B2; BD Biosciences), and anti-CD25 (clone 2A3; BD Biosciences); Brilliant Violet 421–conjugated anti-CD25 (clone BC96; Biolegend) and anti-CXCR3 (clone G025H7; Biolegend); and PE‐Cy7‐conjugated anti-CCR4 (clone TG6/CCR4; Biolegend). After surface staining, the cells were fixed and permeabilized with a Foxp3/transcription factor staining buffer set (eBioscience, San Diego, CA, USA) and intracellular staining for FOXP3 and CTLA-4 was performed using PE‐conjugated anti-*FOXP3* (clone PCH101, eBioscience) and biotin‐conjugated anti-CTLA‐4 (clone BNI3, eBioscience) + PE‐conjugated streptavidin (BD Biosciences). The T cell subsets were gated according to the gating strategy previously published in Aldridge *et al* [37] and also shown in **S2 Fig**. The phenotypes of defined T cell subsets were confirmed by lineage specifying transcription factor expression analysis by rt-qPCR and cytokine secretion analysis by Cytometric Bead Array (BD Biosciences) as previously shown [36]. Small aliquots of fresh blood were used for cell counts of total populations using BD TruCOUNT (Absolute Counting Tubes; BD Biosciences). Stained samples were analyzed using FACSCanto II equipped with FACS Diva software (BD Biosciences) and the resulting data were analyzed with FlowJo software (Tree Star, Ashland, OR, USA).

### Bone densitometry

All patients recruited at the rheumatology clinic in Gothenburg were asked to participate in dual-energy X-ray absorptiometry (DXA) and high resolution peripheral quantitative computed tomography (HR‐pQCT) measurements. 46 of the ueRA patients consented to undergo the analyses. Clinical characteristics of this patient subgroup is shown in **S1 Table**.

Bone mineral density, aBMD (g cm^−2^), was measured at baseline of the study at the total hip, femoral neck and lumbar spine (L_1_–L_4_) in 46 ueRA patients using the Hologic Discovery A (S/N 86491) device (Waltham, MA, USA). The coefficient of variation (CV) for these measurements were 0.8% (total hip), 1.3% (femoral neck) and 0.7% (spine).

Volumetric BMD (vBMD) and bone microarchitecture was measured at the lower leg (tibia) on the same side as the non-dominant arm using a high‐resolution 3D HR‐pQCT device (XtremeCT; Scanco Medical AG, Brüttisellen, Switzerland), according to a previously described protocol [38,39]. In short, the tibia was measured at the standard measuring site recommended by the manufacturer (ultradistal). The first image was acquired at 22.5 mm from the reference line (i.e. a line placed at the articular plateau by the operator). A total of 110 cross‐sectional images were obtained with an isotropic resolution of 82 μm resulting in a three‐dimensional model of the bone. Each three‐dimensional model (110 images) took 3 min of scan time to obtain, and the effective dose was 3 μSv. Quality assessments of the images was performed and graded from 1 to 5, according to the recommendation provided by the manufacturer (Scanco Medical AG), in which 1 to 3 were regarded as acceptable quality and 4 to 5 as unacceptable quality. Only images with quality 1 to 3 were processed further. Each site was analyzed according to the standard HR‐pQCT protocol yielding parameters: trabecular bone volume fraction (BV/TV, the trabecular bone volume to total volume ratio (BV/TV) was derived from the BMD of the trabecular VOI (Tb.vBMD) and making the assumption that compact bone has a matrix mineral density of 1200 mg hydroxyapatite (HA)/cm^3^, whereas the marrow background is equivalent to 0 mg HA/cm^3^), trabecular number (Tb.N, mm^–1^, inverse of the mean spacing of the mid‐axes), trabecular thickness (Tb.Th, mm, (BV/TV)/Tb.N), cortical volumetric BMD (mg/cm^3^), cortical area (mm^2^), and total volumetric BMD (mg/cm^3^). The CVs for measurement of trabecular parameters were 0.8% to 2.6% and the CVs for measurements of cortical parameters were 0.1% to 0.9%.

### Statistical analysis

Statistical analyses were performed using SPSS (version 25; SPSS, Inc., Chicago, IL, USA), GraphPad Prism 8 (La Jolla, CA, USA) and SIMCA 15 software (Umetrics, Umeå, Sweden). Two-tailed Mann-Whitney U test was used for comparison between two groups with non-Gaussian distribution. Multivariate factor OPLS analysis was used to analyze the associations between intermediate monocytes and T-cell subsets. All data were scaled to unit variance by dividing each variable by 1/standard deviation, so that all the variables were given equal weight regardless of their absolute value. The quality of the OPLS models was assessed based on the parameters R2 (i.e., how well the variation of the variables is explained by the model), and Q2 (i.e., how well a variable can be predicted by the model). The variables that contributed most to the OPLS models were further analyzed by univariate analysis. Univariate correlations were performed using two-tailed Spearman’s Rank-Order Correlation. Linear regression models were performed and presented with unstandardized beta values. All non‐normally distributed variables were log‐transformed for inclusion in these linear regressions and the models were adjusted for age, sex and BMI, as described in the legends.

## Results

### Clinical characteristics of ueRA patients

To determine whether the frequency of circulating monocytes correlate with parameters of bone density or microarchitecture in newly diagnosed RA patients, a well-defined study population of ueRA patients was used. Clinical characteristics of the 78 ueRA patients and 29 HC are shown in **Table 1**. The ueRA study population have the characteristics of a typical early RA cohort: 71% of the patients were female and 83.3% were ACPA-positive while 73.1% were RF-positive. The median score of disease activity parameters of the ueRA patients were classified as high (DAS28-ESR>5.1 and CDAI>22).

### No difference in the frequency of classical-, intermediate- or non-classical monocytes between ueRA patients and HC

**Fig 1:**
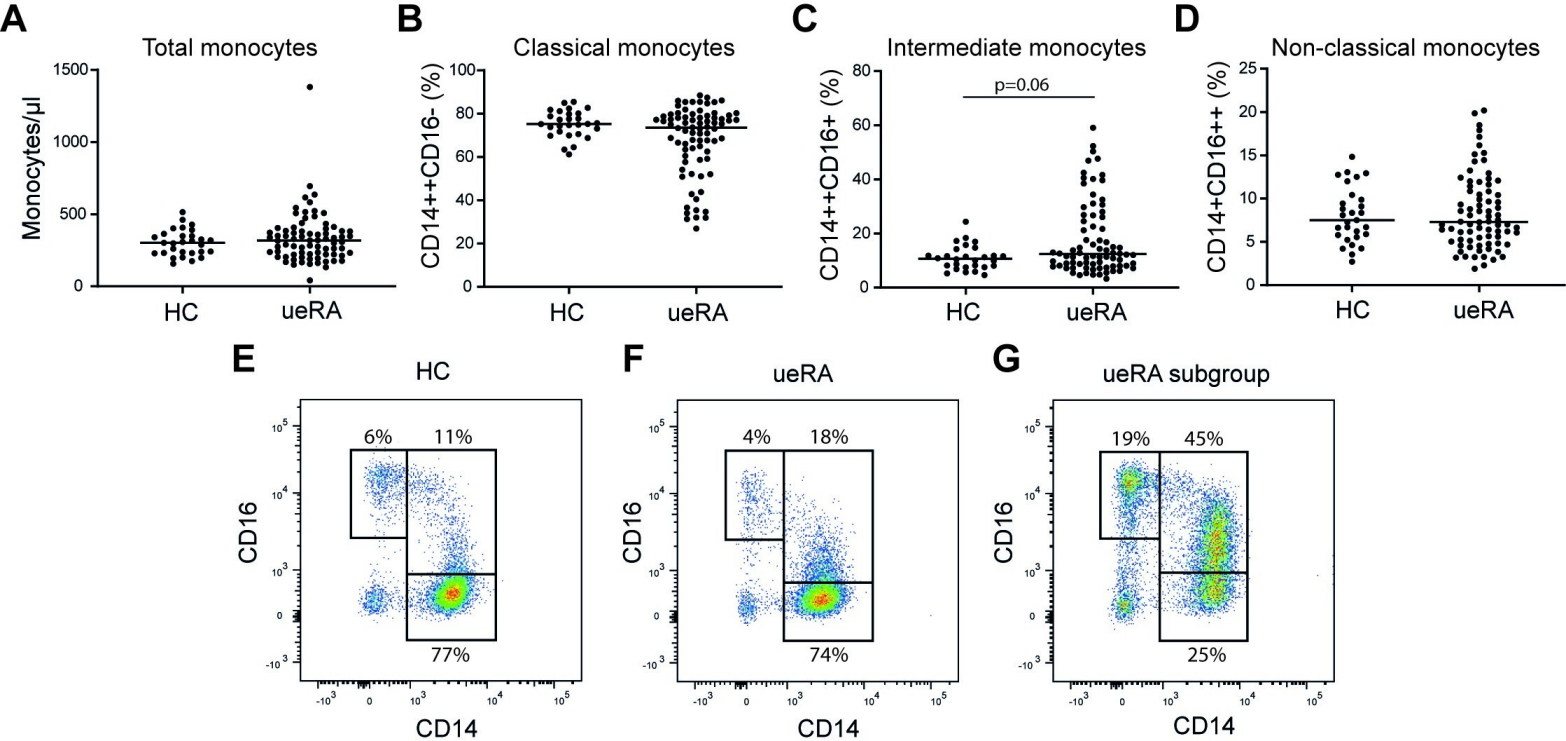
Flow cytometric characterization of the monocyte subpopulations in peripheral blood from HC and ueRA patients. (**A**) Total number of monocytes per μl in HC and ueRA patients measured with TruCount. (**B**) Percentage of classical monocytes (CD14^++^CD16^-^), (**C**) intermediate monocytes (CD14^++^CD16^+^) and (**D**) non-classical monocytes (CD14^+^CD16^++^). (**E**) Representative FACS-image from a HC, (**F**) an ueRA patient with average level of intermediate monocytes, (**G**) an ueRA patient in the subgroup with elevated level of intermediate monocytes. Each data point represents an individual subject. Horizontal bars indicate the median. Statistical analysis: Mann-Whitney U test.

The total number of circulating monocytes and the distribution of the three monocyte subsets were measured in peripheral blood from ueRA patients and HC using flow cytometry. The total number of monocytes per microliter was similar in ueRA patients and HC (**Fig 1A**). Also, the frequency of classical monocytes did not differ between the groups (p = 0.11) but a trend towards increased frequency of intermediate monocytes in ueRA patients was found (p = 0.06) (**Fig 1B, 1C, 1E and 1F**). Although the majority of the ueRA patients had frequencies of intermediate monocytes comparable to the HC, a subgroup of the patients displayed highly elevated levels of this subset. **Fig 1G** shows a representative image of a flow cytometry dot plot from a patient in the subgroup with elevated frequency of intermediate monocytes. The opposite pattern was seen in the distribution of the classical monocytes, where a subgroup of the patients had reduced levels of this population. No difference between ueRA and HC was found in the non-classical monocyte population (p = 0.95) (**Fig 1D, 1E and 1F**). Altogether, these findings show that the proportions of intermediate monocytes are not significantly elevated in patients with untreated early RA.

### Proportions of CXCR3^+^ Th17 cells correlate positively with the frequency of intermediate monocytes in ueRA patients

**Fig 2:**
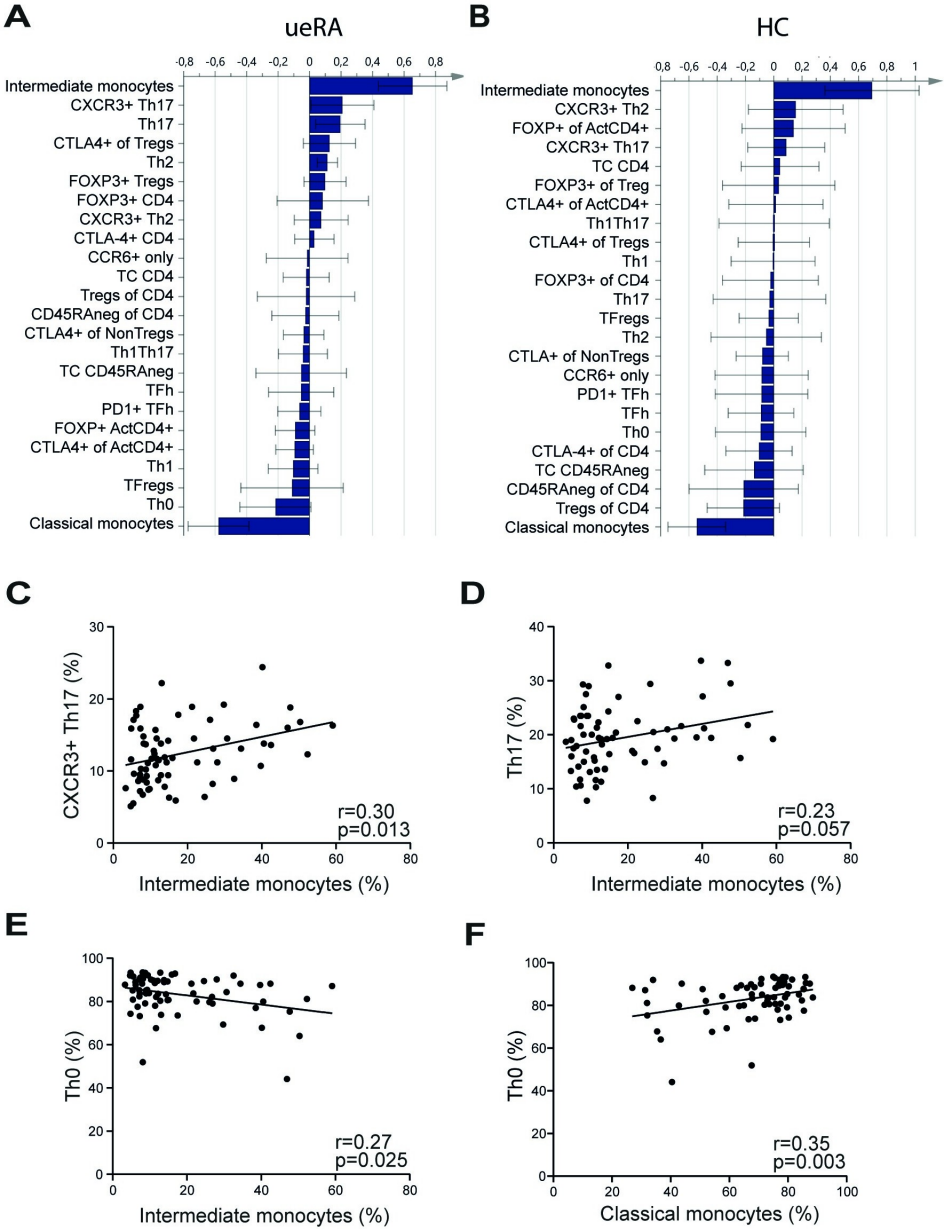
Associations between monocyte and T cell subset proportions in ueRA patients. (**A**-**B**) OPLS column loading plot depicting the association between the proportions of monocyte subsets and T cell subsets in (**A**) ueRA patients and in (**B**) HC. The absolute number of CD4^+^ cells (TC CD4) and CD4^+^CD45RA^-^ cells (TC CD45neg) per unit blood was also included in the analysis. (**C**-**E**) Scatter plots presenting correlations between the frequency of the intermediate monocyte subset and (**C**) CXCR3^+^Th17, (**D**) Th17, and (**E**) Th0 subset proportions. (**F**) Correlation between classical monocytes and Th0. Each data point represents an individual subject. Statistical analysis in **C-F**: Spearman’s rank correlation, r: Spearman’s correlation coefficient.

T cells, and Th17 cells in particular, have been shown to play an important role in various inflammatory conditions including RA, and for osteoporosis [40]. Therefore, we explored associations between intermediate monocytes and a comprehensive panel of circulating T helper cell subsets in this cohort of ueRA patients using multivariate OPLS analysis. The OPLS loading plot displays T cell subsets with the strongest association to the intermediate monocytes. The positively related variables are represented by bars pointing in the same direction as the intermediate monocytes, whereas variables pointing in the opposite direction are inversely associated. CXCR3^+^ Th17 cells and Th17 cells indeed showed the strongest association with intermediate monocytes, whereas Th0 cells most strongly associated with classical monocytes (**Fig 2A**). We also investigated associations between intermediate monocytes and the T cell populations in HC using multivariate OPLS analysis (**Fig 2B**). Although intermediate monocytes associated most strongly with CXCR3^+^ Th2 cells and classical monocytes were closest related with Tregs of CD4 in HC, univariate analysis revealed that none of these correlations were significant (**S3 Fig**).

We further investigated the correlations between intermediate monocytes and CXCR3^+^ Th17 or Th17 cells using univariate analysis (**Fig 2C and 2D**), and found a significant positive correlation with Th17 cells expressing CXCR3 (a chemokine receptor important for infiltration into inflammatory sites [41]), while Th17 did not correlate significantly. In addition, there was a significant negative correlation between Th0 cells and intermediate monocytes, whereas classical monocytes correlated positively with this T cell subset (**Fig 2E and 2F**). In summary, these findings suggest a relation between CXCR3^+^ Th17 cells and intermediate monocytes in newly diagnosed RA patients but not in HC.

### Bone densitometry analysis show no difference in bone density of ueRA patients compared to reference values

DXA and HR-pQCT and were performed on a subpopulation of the ueRA patients (n = 46) to assess bone mineral density (BMD) and bone microarchitecture. The basal characteristics revealed by DXA including T-score/Z-score of the ueRA subpopulation is shown in **S1 Table**. The mean Z-score (comparison of the patient’s bone density with that of an average person of the same age and sex) of the femoral hip of the patients were 0.031±0.95. Thus, bone analysis of ueRA patients reveal no difference in bone density when compared to age- and sex matched reference values.

### Circulating intermediate monocytes or CXCR3^+^ Th17 cells are not associated with bone characteristics

Linear regression was used to investigate whether circulating intermediate monocytes or CXCR3^+^ Th17 cells could be used to predict parameters of bone characteristics in ueRA patients and the model was adjusted for the covariates age, sex and BMI. No significant associations were found between the intermediate monocytes and cortical or trabecular bone parameters assessed by HR-pQCT **(Table 2)**, nor were any associations found between the intermediate monocyte subset and BMD of hip or spine measured with DXA **(S2 Table)**. Further, CXCR3^+^ Th17 did not associate with any of the bone parameters measured with HR-pQCT and DXA **(Tables 2** and **S2)**. In summary, these data indicate that neither the frequency of circulating intermediate monocytes nor the frequency of CXCR3^+^ Th17 cells are associated with effects on bone in early RA.

**Table 2:** Neither the frequency of circulating intermediate monocytes nor the frequency of CXCR3^+^ Th17 cells are associated with bone characteristics measured by HR-pQCT.

HR-pQCT	Intermediate monocytes		CXCR3+Th17	
	B	*p*	β	*p*
Tot. volumetric bone density (mg cm^−3^)	‒16.48	0.56	45.61	0.35
Cortical vBMD (mg cm^−3^)	‒24.53	0.53	41.05	0.53
Cortical area (mm^2^)	‒12.03	0.35	7.80	0.72
Trabecular bone volume fraction (%)	0.008	0.53	0.023	0.37
Trabecular number (mm^−1^)	0.247	0.14	0.230	0.42
Trabecular thickness (mm)	0.004	0.64	0.003	0.82

Linear regression analysis with bone parameters (dependent variable) and intermediate monocytes, CXCR3+Th17 (independent variable). vBMD volumetric bone mineral density. β are unstandardized coefficients. Significant p values are shown in bold typeface. Adjusted for age, sex and BMI. N = 46.

### Frequency of intermediate monocytes correlate with age in ueRA patients

**Fig 3:**
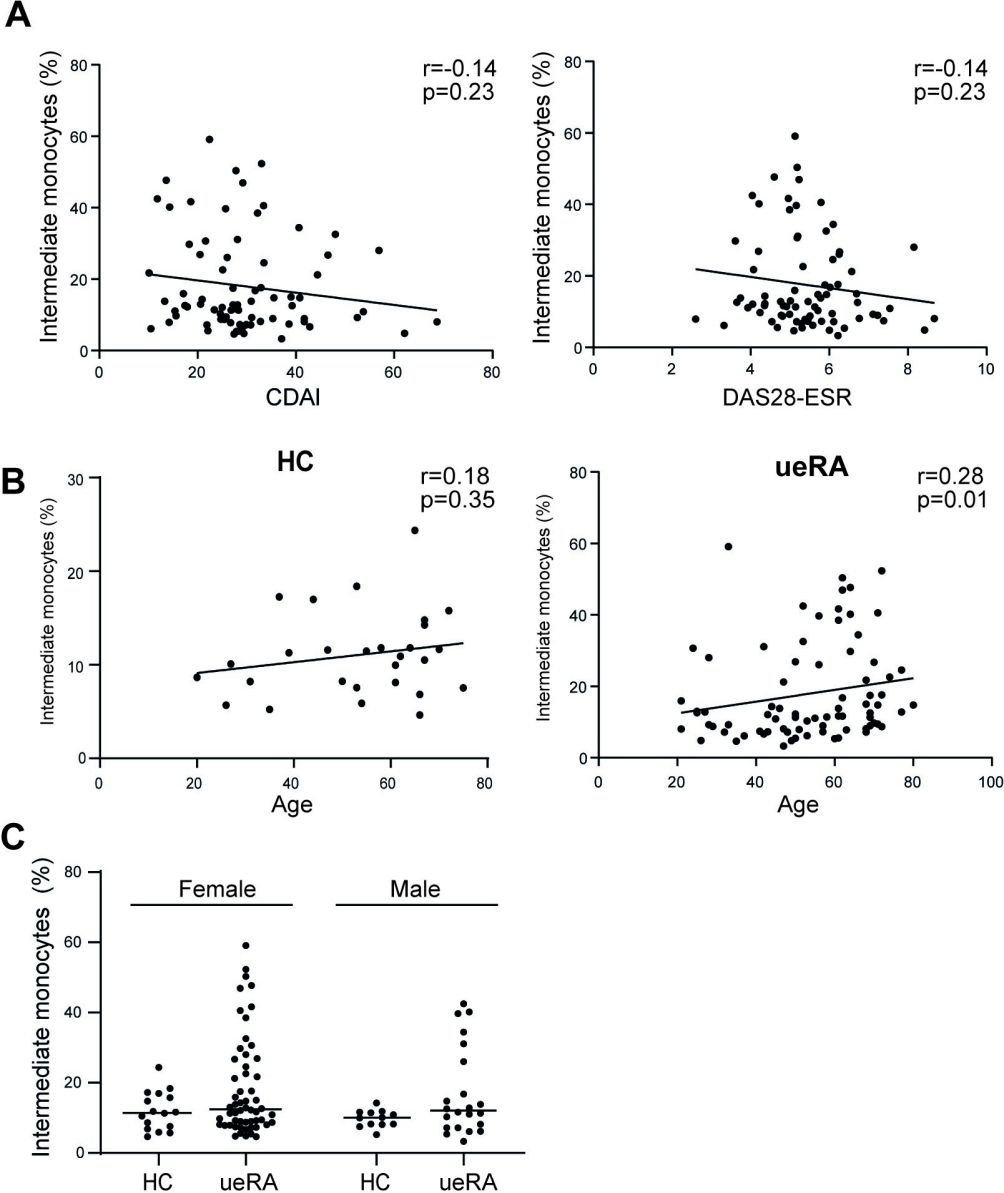
Relations between the proportion of intermediate monocytes and disease activity, age and sex. (**A**) Correlation between intermediate monocytes and CDAI or DAS28-ESR. (**B**) Correlation between intermediate monocytes and age in HC and ueRA. (**C**) Distribution of intermediate monocytes in females and males. Each data point represents an individual subject. Horizontal bars indicate the median. Statistical analysis in (**A-B**): Spearman’s rank correlation, r: Spearman’s correlation coefficient; and (**C)**: Mann-Whitney U test.

Next we examined correlations between intermediate monocytes and disease activity, age or sex using univariate analyses. No significant correlations were found between intermediate monocytes and any disease parameters (**Fig 3A**). A weak positive correlation between age and intermediate monocytes was detected in ueRA patients, but not in HC (**Fig 3B**). No differences in the frequency of circulating intermediate monocytes between ueRA patients and HC were found in either females or males when subdivided (**Fig 3C**), and both female and male ueRA patients displayed subgroups with increased frequency of intermediate monocytes (**Fig 3C**). Altogether, these data show that the frequency of circulating intermediate monocytes does not correlate with disease activity, but does correlate with age in early RA.

## Discussion

In this study, we investigated the role of intermediate monocytes in RA and their relation to T cell subsets and bone parameters in untreated newly diagnosed RA patients. We show that the proportions of intermediate monocytes are not significantly elevated in peripheral blood of ueRA patients compared to HC. There is a significant positive correlation between intermediate monocytes and the frequency of CXCR3^+^ Th17 cells in ueRA patients that is not found in HC, but neither intermediate monocytes nor CXCR3^+^ Th17 cells were associated with parameters of bone density or bone microarchitecture in ueRA patients. The majority of the ueRA patients had similar frequencies of intermediate monocytes as HC, whereas a subgroup of the patients had elevated levels of this subset, which resulted in an overall trend towards increased frequency of intermediate monocytes in ueRA patients compared to HCs (p = 0.06).

Previous publications report discrepant results regarding the levels of intermediate monocytes in RA patients compared to HC. While Yoon *et al* showed a distribution pattern of the intermediate monocyte population similar to what we show in this study, with a subpopulation of RA patients displaying increased levels of intermediate monocytes [21]; some of the other studies, *e*.*g*. Rossol *et al* and Tsukamoto *et al*, show larger significant differences between RA patients and HC in intermediate monocyte levels [19,20]. Treatment with methotrexate has been reported to decrease the intermediate monocyte subset [20,42], while glucocorticoid treatment specifically induces this subset [43]. In addition, the levels of intermediate monocytes have been reported to correlate positively with disease duration [17]. The patients in our study were both corticosteroid- and DMARD-naïve as well as newly diagnosed, which is in contrast to the study by Rossol *et al* [14], where the patient group had a mean disease duration of 15 years and were subjected to various treatments. The patients included in the study by Tsukamoto and coworkers [15] were DMARD-naïve, but the disease duration prior to inclusion in that study was on average 42 months, in comparison to the patients in this study who had a mean self-reported symptom duration of 6 months. We show that proportions of intermediate monocytes correlate positively with age in ueRA patients but not in HC, indicating the importance of age in the study groups. In this study, the median age was similar in the patient group and HC (ueRA: 56 years *vs* HC: 58 years, while in the study of Tsukamoto *et al*, the mean age in controls and patients differed (RA: 60 years *vs* HC: 49 years) [20]. The fact that intermediate monocytes correlated positively with age in ueRA but not in HC in this study, could have resulted in increased differences between ueRA patients and HC in proportions of intermediate monocytes, particularly among the ueRA patients and age-matched HC of the highest age group. However, no difference in proportions of intermediate monocytes between ueRA patients and HC was reported in this study. To conclude, the patients in our study represent a homogenous treatment-naïve group with a short duration of self-reported symptoms and we speculate that at later time points in RA disease development the patients may show elevated levels of circulating intermediate monocytes.

No previous studies have explored associations between monocyte subsets and a comprehensive panel of CD4^+^ helper and regulatory T cell subsets in peripheral blood of RA patients. Here, we investigated this using multivariate analysis and found a significant positive correlation between frequencies of circulating intermediate monocytes and CXCR3^+^ Th17 cells from ueRA patients. In HC, there were no significant correlations between intermediate monocytes and any of the T cell subsets indicating that this finding is specific to RA disease. However, previously published reports with the same ueRA patient cohort showed that the dominating circulating T cell subsets in ueRA patients were Th2 and Th17 cells, whereas CXCR3^+^ Th17 showed less association with disease [36], but CXCL10 (one of the ligands of CXCR3) was shown to correlate positively with clinical disease activity in the ueRA patients [31]. Blocking of CXCR3 has previously been shown exert anti-arthritic effects via inhibition of chemokines and inflammatory mediators in mice with collagen-induced arthritis [44]. CXCR3 is known to affect the migration of immune cells into inflammatory sites, *e*.*g* inflamed joints in RA [45,46]. In line with this, Aldridge *el al*, recently reported that the frequency of CXCR3^+^Th17 cells is higher in synovial fluid than in peripheral blood of RA patients, indicating that these cells migrate from blood to the inflamed joints [47]. CXCL10 was shown to stimulate the expression of the osteoclastogenic cytokines RANKL, TNFα, and IL-6 in CD4^+^ T cells [48]. Furthermore, a recent publication showed that CXCR3 expression on osteoclast precursors was involved in homing to bone resorption sites [49]. Whether CXCR3^+^ Th17 cells are also recruited to bone resorption sites and how they might affect osteoclastogenesis by RANKL production is not known, and future studies are needed to elucidate the relation between CXCR3^+^ Th17 cells, intermediate monocytes and bone remodeling. Previously, Rossol *et al* have shown that Th17 cells correlate with the intermediate monocyte subset in patients with established RA [19] but in this study only a trend was found, which might be explained by differences in disease duration and treatments in this cohort compared to that in Rossol *et al*. To summarize, these findings show for the first time an association between proportions of intermediate monocytes and CXCR3^+^ Th17 cells in RA patients with early untreated disease.

Individuals with RA have an increased risk of developing osteoporosis, which is related to increased disease activity [50,51]. However, there are only few studies that have investigated the osteoclastogenic potential of intermediate monocytes [15,52] and so far it is not known whether increased frequencies of this monocyte population results in bone loss in RA patients. Therefore, we investigated whether proportions of circulating intermediate monocytes are associated with bone characteristics in early RA. In addition to bone density measurement with the commonly used DXA method, we also assessed volumetric bone mineral density and bone microarchitecture by the sensitive imaging method HR-pQCT. We show that proportions of intermediate monocytes do not correlate with parameters of bone density or microarchitecture in the early stages of RA using any of the bone densitometry techniques. As CXCR3 expression has been connected to local bone loss in animal models [48,53], we also investigated associations between CXCR3^+^ Th17 cells and bone measurements in ueRA, but no associations were found. In other studies, bone loss has been described within the first year after RA diagnosis [54,55], but in this study the ueRA patients did not yet display decreased bone density when compared to age- and sex-matched reference values. Hence, we speculate that to fully explore the potential of intermediate monocytes or CXCR3^+^ Th17 cells to drive bone loss in RA, future longitudinal studies in patient groups with longer disease duration and more progressed bone loss are required.

Finally, we investigated whether increased levels of intermediate monocytes in ueRA would result in a more severe disease, but found no correlations between intermediate monocytes and any parameters for disease activity. This is in contrast to Tsukamoto *et al* who showed a positive correlation between disease activity and intermediate monocytes [20]. However, as mentioned above, the patients in that study had a longer disease duration compared to the patients in this study, which might explain this discrepancy. Also, associations between the T cell subset proportions and clinical parameters have been investigated previously in this cohort of RA patients by Pandya *et al*, but no associations were found [36].

There are limitations to this study. First, the number of patients who performed bone densitometry was relatively small, however the r^2^ value of the dependent variable (CXCR3^+^ Th17 or intermediate monocytes), was very low (between 0.006 and 0.059) without the inclusion of BMI, sex and age. Thus, it is unlikely that a larger population would result in a large r^2^ and significant predictions. Second, our data are snapshot measurements of monocyte and T cell levels in the circulation of HC and ueRA patients and do not reflect the turnover of the subsets. Third, we did not investigate monocyte and T cell subsets in joint synovial fluid, but only in peripheral blood, which might not reflect the rate and extent of infiltration of these cell types into inflamed joints.

## Conclusions

To our knowledge, this is the first study evaluating the role of intermediate monocytes and a broad spectrum of T cell subsets on bone density and bone microarchitecture in newly diagnosed untreated RA patients. We used multivariate analysis to examine the associations between monocytes and T cell subsets in peripheral blood of the ueRA patients. We show that the frequency of circulating intermediate monocytes is not significantly increased in patients with ueRA compared to HC. The intermediate monocyte population correlate positively with CXCR3^+^ Th17 cells in ueRA patients but not in HC. However, neither proportions of intermediate monocytes nor CXCR3^+^ Th17 cells are associated with bone density or bone microarchitecture measurements. Thus, our results suggest that in early RA, the intermediate monocytes do not correlate with bone characteristics, despite positive correlation with circulating CXCR3^+^ Th17 cells. Future longitudinal studies in patients with longer disease duration are required to fully explore the potential of intermediate monocytes to drive bone loss in RA.

## Supporting information

S1 Fig Gating strategy of the three monocyte subsets. **(1)** Initially monocytes were gated according to forward scatter area (FSC-A) and side scatter area (SSC-A) characteristics. **(2)** Doublet discrimination was done using FCS-A and FCS-Height (FSH-H). **(3)** The monocyte populations were subdivided based upon expression of CD14 and CD16. Three monocyte subsets were distinguished: classical monocytes (CD14^++^CD16^-^), intermediate monocytes (CD14^++^CD16^+^) and non-classical monocytes (CD14^+^CD16^++^).
(PDF)

S2 Fig Gating strategy of CD4+ T cell subsets previously published in Aldridge *et al* (Arthritis Research and Therapy 2018, 20:150). The gating strategy (from a representative female RA patient) was as follows: (a) singlet PBMCs were gated for lymphocytes and then further gated for CD4^+^ T cells. CD4^+^ cells where then divided into naïve (CD45RA^+^) and memory (CD45RA^neg^) subsets. From naïve cells, CCR4^neg^CCR6^neg^CXCR3^neg^ cells were defined as Th0. Memory cells were divided into four subsets based on CCR4 and CCR6 expression, each of which was the further divided based on CXCR3 expression; Th1 (CCR4^neg^CCR6^neg^CXCR3^+^), Th2 (CCR4^+^CCR6^neg^CXCR3^neg^), CXCR3^+^Th2 (CCR4^+^CCR6^neg^CXCR3^+^), Th17 (CCR4^+^CCR6^+^CXCR3^neg^), CXCR3^+^Th17 (CCR4^+^CCR6^+^CXCR3^+^), Th1Th17 (CCR4^neg^CCR6^+^CXCR3^+^), and CCR6^+^ only (CCR4^neg^CCR6^+^CXCR3^neg^). (b) The cutoff for CTLA-4 positivity on CD4^+^ T cells were determined using fluorescence minus one (FMO) and cutoff for FOXP3 positivity in CD4^+^ cells was based on FOXP3 expression in CD25^neg^ gated CD4^+^ cells. (c) Regulatory T cells (Tregs) were defined by CD25^+^CD127^low^ expression, while the remaining cells were defined as non-Tregs. CXCR5^+^ Tregs were defined as follicular regulatory T cells (TFregs) and CXCR5^+^ non-Tregs as follicular helper T cells (TFh).
(PDF)

S3 Fig Associations between monocyte and T cell subset proportions in HC. Scatter plots presenting correlations between (**A**) frequency of the classical monocyte subset and Tregs of CD4, and (**B**) frequency of the intermediate monocyte subset and CXCR3+Th2. Each data point represents an individual subject. Statistical analysis: Spearman’s rank correlation, r: Spearman’s correlation coefficient.
(PDF)

S1 Table Clinical characteristic of the subpopulation of ueRA patients undergoing bone densitometry. (PDF)

S2 Table The frequencies of intermediate monocytes and CXCR3^+^ Th17 cells are not associated with BMD measured by DXA. (PDF)
