# Supplementary material for: Intermediate monocytes correlate with CXCR3+ Th17 cells but not with bone characteristics in untreated early rheumatoid arthritis
Source: PLoS One. 2021 Mar 26;16(3):e0249205. doi: 10.1371/journal.pone.0249205 (PMC7996983; doi:10.1371/journal.pone.0249205)
Supplement: S1 Fig — (1) Initially monocytes were gated according to forward scatter area (FSC-A) and side scatter area (SSC-A) characteristics. (2) Doublet discrimination was done using FCS-A and FCS-Height (FSH-H). (3) The monocyte populations were subdivided based upon expression of CD14 and CD16. Three monocyte subsets were distinguished: classical monocytes (CD14++CD16-), intermediate monocytes (CD14++CD16+) and non-classical monocytes (CD14+CD16++). (PDF) [file pone.0249205.s001.pdf]

S1 Figure

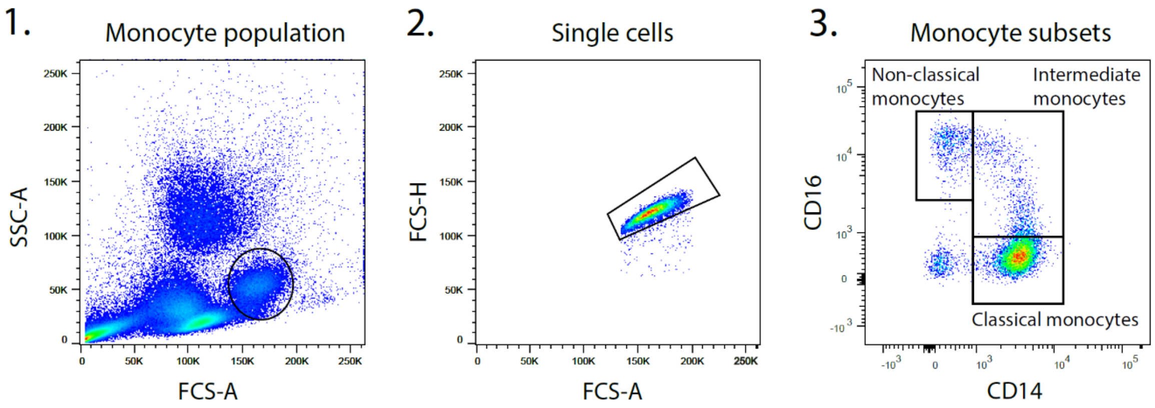

S1 Fig. Gating strategy of the three monocyte subsets. (1) Initially monocytes were gated according to their forward scatter area (FSC-A) and side scatter area (SSC-A) characteristics. (2) Doublet discrimination was done using FCS-A and FCS-Height (FSH-H). (3) The monocyte populations were then subdivided based upon their expression of CD14 and CD16. Three monocyte subsets were distinguished: classical monocytes ($CD14^{++}CD16^{-}$), intermediate monocytes ($CD14^{++}CD16^{+}$) and non-classical monocytes ($CD14^{+}CD16^{++}$).
