# Supplementary material for: Intermediate monocytes correlate with CXCR3+ Th17 cells but not with bone characteristics in untreated early rheumatoid arthritis
Source: PLoS One. 2021 Mar 26;16(3):e0249205. doi: 10.1371/journal.pone.0249205 (PMC7996983; doi:10.1371/journal.pone.0249205)
Supplement: S2 Fig — The gating strategy (from a representative female RA patient) was as follows: (a) singlet PBMCs were gated for lymphocytes and then further gated for CD4+ T cells. CD4+ cells where then divided into naïve (CD45RA+) and memory (CD45RAneg) subsets. From naïve cells, CCR4negCCR6negCXCR3neg cells were defined as Th0. Memory cells were divided into four subsets based on CCR4 and CCR6 expression, each of which was the further divided based on CXCR3 expression; Th1 (CCR4negCCR6negCXCR3+), Th2 (CCR4+CCR6negCXCR3neg), CXCR3+Th2 (CCR4+CCR6negCXCR3+), Th17 (CCR4+CCR6+CXCR3neg), CXCR3+Th17 (CCR4+CCR6+CXCR3+), Th1Th17 (CCR4negCCR6+CXCR3+), and CCR6+ only (CCR4negCCR6+CXCR3neg). (b) The cutoff for CTLA-4 positivity on CD4+ T cells were determined using fluorescence minus one (FMO) and cutoff for FOXP3 positivity in CD4+ cells was based on FOXP3 expression in CD25neg gated CD4+ cells. (c) Regulatory T cells (Tregs) were defined by CD25+CD127low expression, while the remaining cells were defined as non-Tregs. CXCR5+ Tregs were defined as follicular regulatory T cells (TFregs) and CXCR5+ non-Tregs as follicular helper T cells (TFh). (PDF) [file pone.0249205.s002.pdf]

S2 Figure

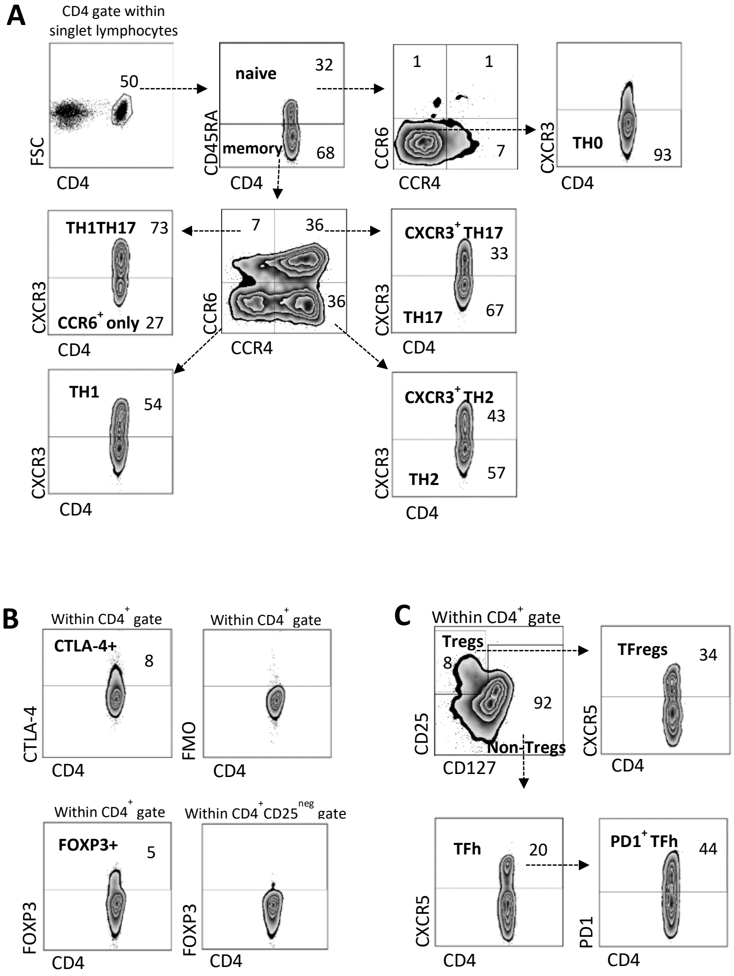

Fig S2. Gating strategy of CD4+ T cell subsets previously published in Aldridge *et al* (Arthritis Research and Therapy 2018, 20:150). The gating strategy (from a representative female RA patient) was as follows: (a) singlet PBMCs were gated for lymphocytes and then further gated for $CD4^+$ T cells. $CD4^+$ cells where then divided into naïve ($CD45RA^+$) and memory ($CD45RA^{neg}$) subsets. From naïve cells, $CCR4^{neg}CCR6^{neg}CXCR3^{neg}$ cells were defined as Th0. Memory cells were divided into four subsets based on CCR4 and CCR6 expression, each of which was the further divided based on CXCR3 expression; Th1 ($CCR4^{neg}CCR6^{neg}CXCR3^+$), Th2 ($CCR4^+CCR6^{neg}CXCR3^{neg}$), $CXCR3^+$Th2 ($CCR4^+CCR6^{neg}CXCR3^+$), Th17 ($CCR4^+CCR6^+CXCR3^{neg}$), $CXCR3^+$Th17 ($CCR4^+CCR6^+CXCR3^+$), Th1Th17 ($CCR4^{neg}CCR6^+CXCR3^+$), and $CCR6^+$ only ($CCR4^{neg}CCR6^+CXCR3^{neg}$). (b) The cutoff for CTLA-4 positivity on $CD4^+$ T cells were determined using fluorescence minus one (FMO) and cutoff for FOXP3 positivity in $CD4^+$ cells was based on FOXP3 expression in $CD25^{neg}$ gated $CD4^+$ cells. (c) Regulatory T cells (Tregs) were defined by $CD25^+CD127^{low}$ expression, while the remaining cells were defined as non-Tregs. $CXCR5^+$ Tregs were defined as follicular regulatory T cells (TFregs) and $CXCR5^+$ non-Tregs as follicular helper T cells (TFh).
