# Supplementary material for: Intermediate monocytes correlate with CXCR3+ Th17 cells but not with bone characteristics in untreated early rheumatoid arthritis
Source: PLoS One. 2021 Mar 26;16(3):e0249205. doi: 10.1371/journal.pone.0249205 (PMC7996983; doi:10.1371/journal.pone.0249205)
Supplement: S3 Fig — Scatter plots presenting correlations between (A) frequency of the classical monocyte subset and Tregs of CD4, and (B) frequency of the intermediate monocyte subset and CXCR3+Th2. Each data point represents an individual subject. Statistical analysis: Spearman’s rank correlation, r: Spearman’s correlation coefficient. (PDF) [file pone.0249205.s003.pdf]

S3 Figure

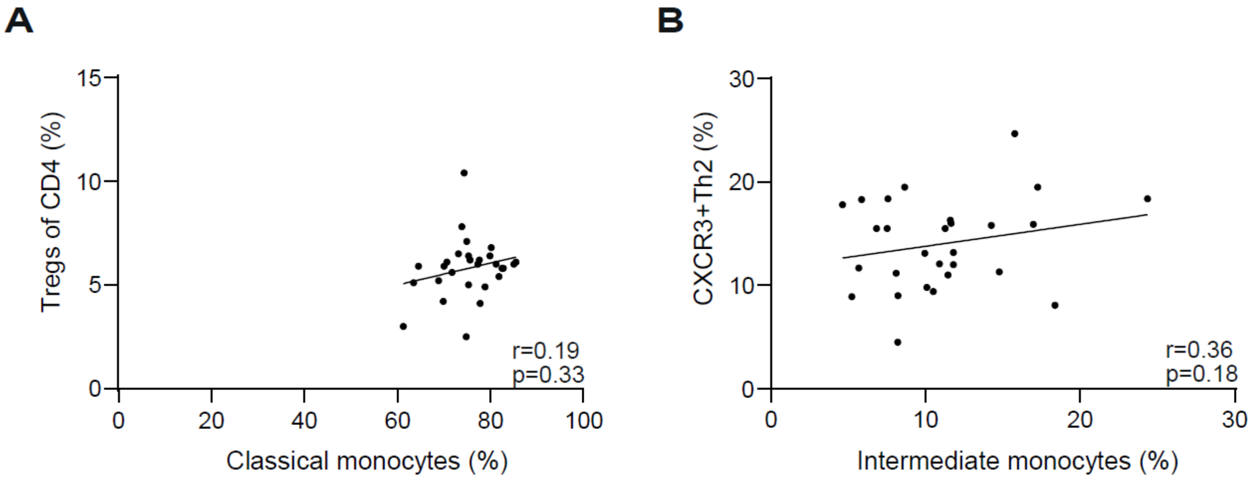

Fig S3. **Associations between monocyte and T cell subset proportions in HC.** Scatter plots presenting correlations between (**A**) frequency of the classical monocyte subset and Tregs of CD4, and (**B**) frequency of the intermediate monocyte subset and CXCR3+Th2. Each data point represents an individual subject. Statistical analysis: Spearman’s rank correlation, r: Spearman's correlation coefficient.
