# Supplementary material for: Intermediate monocytes correlate with CXCR3+ Th17 cells but not with bone characteristics in untreated early rheumatoid arthritis
Source: PLoS One. 2021 Mar 26;16(3):e0249205. doi: 10.1371/journal.pone.0249205 (PMC7996983; doi:10.1371/journal.pone.0249205)
Supplement: S1 Table — (PDF) [file pone.0249205.s004.pdf]

**S1 Table.** Clinical characteristic of the subpopulation of ueRA patients undergoing bone densitometry

| | **ueRA patients (*n* = 46)** |
|---|---|
| Age, years | 56.5 (21-80) |
| Female, n (%) | 33 (71.7) |
| Self-reported symptom duration, months | 5 (1-23) |
| CRP, mg/L | 7 (0.5-180) |
| ESR, mm/hour | 23 (5-101) |
| SJC66 | 10 (3-28) |
| TJC68 | 11 (2-35) |
| SJC28 | 7.5 (2-24) |
| TJC28 | 6 (0-27) |
| DAS28-CRP | 4.9 (2.7-8.3) |
| DAS28-ESR | 5.2 (2.6-8.7) |
| CDAI | 27.2 (10.1-68.7) |
| ACPA+, n (%) | 41 (89.1) |
| RF+, n (%) | 31 (67.4) |
| ACPA+ and RF+, *n* (%) | 29 (63.0) |
| ACPA- and RF-, *n* (%) | 3 (6.52) |
| Smoker, n (%)[a] | 4 (8.70) |
| T-score, femural hip, mean (SD)[b] | -0.92 (0.95) |
| > −1.0, n (%) | 20 (50) |
| − 1.0 to − 2.49, n (%) | 18 (45) |
| ≤ − 2.5, n (%) | 2 (5) |
| Z-score, femural hip, mean (SD)[b] | 0.031 (0.95) |
| > −1.0, n (%) | 33 (83) |

Continuous data is presented as median (range). *ACPA* anti-citrullinated protein/peptide antibodies, *CDAI* clinical disease activity index, *CRP* C-reactive protein, *DAS28* disease activity score in 28 joints, *ESR* erythrocyte sedimentation rate, *RF* rheumatoid factor, *SJC 28/66* swollen joint counts of 28/66, *TJC 28/68* tender joint counts of 28/68, arthritis, *NA* not analyzed. [a]Current daily smoker. [b]n=40.
