# Supplementary material for: Intermediate monocytes correlate with CXCR3+ Th17 cells but not with bone characteristics in untreated early rheumatoid arthritis
Source: PLoS One. 2021 Mar 26;16(3):e0249205. doi: 10.1371/journal.pone.0249205 (PMC7996983; doi:10.1371/journal.pone.0249205)
Supplement: S2 Table — (PDF) [file pone.0249205.s005.pdf]

S2 Table. The frequencies of intermediate monocytes and CXCR3$^+$ Th17 cells are not associated with BMD measured by DXA

**DXA**	**Intermediate monocytes**		**CXCR3$^+$Th17**	
	**β**	***p***	**β**	***p***
Femural neck (g cm^{-2})	−0.073	0.22	−0.038	0.70
Hip total (g cm^{-2})	−0.059	0.35	−0.018	0.86
Lumbar spine (g cm^{-2})	−0.087	0.32	−0.113	0.45

Linear regression analysis with bone parameters (dependent variable) and intermediate monocytes (log), CXCR3$^+$ Th17 cells (log) (independent variable). β are unstandardized coefficients. Adjusted for age, sex and BMI. N=44.
